# Shall Army Medical Officers Engage in Private Practice>

**Published:** 1884-09

**Authors:** 

**Affiliations:** Chicago, Ill.


SHALL ARMY MEDICAL OFFICERS ENGAGE IN PRIVATE PRACTICE?

*Sir:*—It is a principle that, in the laws governing a community, the rights and privileges of individuals should not be restricted farther than is necessary to the common good of the whole. To curtail the rights of one class, in the interests of another class, not benefiting the whole, is arbitrary and tyrannical. To restrict the rights of one class of medical men, in the interests of another class, without thereby benefiting the public at large, is a case of this kind. When Dr. Stanton asks that the Army Surgeon at Fort Trumble be prohibited from engaging in civil practice, he asks it, not in the interests of the community, but in his own interest.

The public is not benefited thereby; on the contrary, they would be deprived of the right to call in the Army Surgeon in case they preferred his services to those of Dr. Stanton. The public have a right to select a physician from amongst all those competent and willing to serve it, and any law restricting its privilege in this particular would be an injury and not a benefit. They do not patronize the Army Surgeon for *his* benefit, but for their *own*. The advantage of having the advice of an Army Surgeon is in many parts of the country a very important one, and especially so at small towns and frontier stations, where the local medical talent is not infrequently of inferior quality. People everywhere are entitled to the best medical and surgical advice, which they can procure, and any law restraining them in their choice can only be regarded as an arbitrary injustice.

On the frontier, the Army Surgeon often affords the best, if not the only medical talent at hand, and if he were prohibited from rendering his services the people would be the greatest sufferers. Many instances in proof of this might be cited. If, in the eastern cities, the Army Surgeon who happens to be stationed there is called upon, it is because his services are required or preferred, and so long as he is competent and conducts himself according to the rules of medical practice, governing respectable physicians of the place, it is merely the exercise of a right to which the public is entitled, the curtailing of which would be oppressive and wrong.

That these cities are over-crowded with *so-called* doctors, and that there exists among them a struggle for life, is no argument in favor of prohibiting a class of competent men from giving the benefit of their services to the public when called upon to do so. Instead of crying out in their own interest and to the detriment of the public for the summary suppression of the few who happen to be subject to military law, it would be far more manly to stand upon their own individual merits for success in the contest and trust to the survival of the fittest.

The number of army surgeons stationed at any one time in eastern cities is insignificantly small as compared with the number of civil practitioners, and ought not to be regarded with so much professional jealousy as to call for their extermination, which, if accomplished by military order, would give no perceptible relief to the pressure which exists there. Instead of attempting to inflict a useless and arbitrary wrong upon a class, already subjected to many hardships and poorly paid, it were far more sensible for the civil physicians to direct their energies against the real source of the difficulties of which they have to complain. It is not in efforts to deprive the public of the services of a few scattering individuals, well trained and competent to practice their profession, but by checking the flood of uneducated and unprepared incompetents, armed with diplomas and turned loose amongst them every year, that the evil is to be remedied. The State legislatures should be made to see the folly of chartering in every little town throughout the land, for the issuing of licenses to kill, so-called medical schools, with out any resources or appliances to teach, train or prepare their students to practice the profession into which they are admitted, with full powers and privileges, and, so far as the public can discriminate, on a par with most of the best schools in the land. The man who has devoted his best energies without stint of time or means and acquired in the most favored institutions at home and abroad a liberal literary and medical education must take his place at the bedside with the ignorant clod-hopper whose preparation consists in his having spent a couple of winters, when there was nothing to do on the farm, in a room over a grocery store in some town listening to a few country doctors tell their experience!

They are both doctors, armed alike with diplomas and licenses to practice. The public can discriminate only in point of personal appearance and manners, but not as to professional attainments. Thus the country is over-crowded with incompetents. Doctoring has been reduced to a business, and the practitioner presents himself in whatever shape will bring him the most victims, whether as homœopath, alopath, eclectic, or a mixture of them all, and thus the people are imposed upon and the profession brought into distrust and disgrace. The remedy for all this is to induce state legislators to grant charters only to institutions properly provided with the means and appliances to teach the different branches of medicine and surgery as they should be taught to turn out competent practitioners. There will be numskulls enough even then; but the first step is to close the doors to the enormous abuses now practiced upon public confidence, guarding its interests by giving it better physicians, of which it stands in great need.

AN ARMY SURGEON.

Chicago, Ill., Aug. 20.

